# Impacts of the ENSO Modoki and other Tropical Indo-Pacific Climate-Drivers on African Rainfall

**DOI:** 10.1038/srep16653

**Published:** 2015-11-16

**Authors:** B. Preethi, T. P. Sabin, J. A. Adedoyin, K. Ashok

**Affiliations:** 1 Indian Institute of Tropical Meteorology, Pune 411008, India; 2 Physics Department, University of Botswana, Gaborone, Botswana; 3 University Centre for Earth and Space Sciences, University of Hyderabad 500046, India

## Abstract

The study diagnoses the relative impacts of the four known tropical Indo-Pacific drivers, namely, El Niño Southern Oscillation (ENSO), ENSO Modoki, Indian Ocean Dipole (IOD), and Indian Ocean Basin-wide mode (IOBM) on African seasonal rainfall variability. The canonical El Niño and El Niño Modoki are in general associated with anomalous reduction (enhancement) of rainfall in southern (northern) hemispheric regions during March-May season. However, both the El Niño flavours anomalously reduce the northern hemispheric rainfall during June-September. Interestingly, during boreal spring and summer, in many regions, the Indian Ocean drivers have influences opposite to those from tropical Pacific drivers. On the other hand, during the October-December season, the canonical El Niño and/or positive IOD are associated with an anomalous enhancement of rainfall in the Eastern Africa, while the El Niño Modoki events are associated with an opposite impact. In addition to the Walker circulation changes, the Indo-Pacific drivers influence the African rainfall through modulating jet streams. During boreal summer, the El Niño Modoki and canonical El Niño (positive IOD) tend to weaken (strengthen) the tropical easterly jet, and result in strengthening (weakening) and southward shift of African easterly jet. This anomalously reduces (enhances) rainfall in the tropical north, including Sahelian Africa.

Africa experiences large spatial variability in rainfall, with relatively high mean annual rainfall along equatorial region, poleward extremes, and also over the highland areas such as eastern Africa, Cameroon and Nigeria and coastal sectors of Liberia, Sierra Leone and Guinea; lower annual rainfall is, on the other hand, seen in the arid regions of Sahara desert in subtropical latitudes, Namib desert in the coastal areas of southern Africa and desert areas in equatorial eastern Africa^1^,^2^. The seasonal rainfall maximum transits from south to north depending on the movement of inter tropical convergence zone (ITCZ), from Zimbabwe and central Mozambique during austral summer to Saharan margin during boreal summer. A study by Nicholson^2^ provides the details of rainfall variability over Africa. The different regions of Africa can be observed from the Fig. A1.

Oceanic forcing from different basins, such as the tropical Atlantic^3^,^4^, tropical Pacific^5^,^6^ and Indian Ocean^7^,^8^ plays a dominant role in the interannual variability of rainfall in Africa. The Atlantic, for example, influences especially through its impact on the West African Monsoon system. The rainfall over western region of Africa is directly affected by circulation over the Atlantic and indirectly by Indian Ocean. The tropical Pacific Sea Surface Temperatures (SST) influence the African rainfall directly through “atmospheric bridge” by altering the Walker Circulation, Indian Ocean^9^,^10^, and indirectly through its signature in the tropical Atlantic SST^11^,^12^ and circulation over the Atlantic^13^.

Among the tropical Indo-Pacific climate drivers, the canonical El Niño Southern Oscillation [ENSO]^14^ plays a dominant role in rainfall distribution over various parts of Africa during all the seasons^15^,^16^,^17^. The Indian Ocean Basin Mode [IOBM]^18^, which is an ENSO-induced heat flux anomaly^19^,^20^, influences African climate during boreal winter and spring^21^. In addition, the Indian Ocean Dipole [IOD]^22^ events affect the east African rainfall independent of ENSO especially during the September-November period^8^,^22^. It should be noted that the impact of the ENSO Modoki^23^,^24^, the other ENSO flavour, on African rainfall is not yet understood.

The ENSO Modoki has distinct signatures in SST anomaly (SSTA) and its evolution as compared the canonical ENSO. El Niño (La Niña) Modoki events are characterised with warm (cold) SSTA in the central Pacific flanked on the east and west by cooler (warmer) SSTA^23^,^24^, whereas canonical El Niño (La Niña) refers to anomalous warming (cooling) in the eastern equatorial Pacific Ocean often accompanied by cooler (warmer) SSTA in the western Pacific^14^. The global teleconnections of ENSO Modoki are also different from that of canonical ENSO^23^,^24^. They are opposite to one another, or weaker/stronger or asymmetric, depending on the region and the season of teleconnection. Importantly, a simultaneous occurrence of IOD and canonical ENSO (ENSO Modoki) events results in an interference of individual impacts on the climates of various parts of the world^25^,^26^,^27^,^28^,^29^,^30^,^31^,^32^,^33^. Notably, both the El Niño Modoki and positive IOD events have been increasing in frequency during last three decades^23^,^29^.

Given the interference between teleconnections of the various tropical Indo-Pacific drivers, this study is focused on assessing the relative impact of the known tropical Indo-Pacific drivers, namely ENSO Modoki, canonical ENSO, and IOD/IOBM (depending on the seasons they mainly prevail in), on African rainfall variability during different seasons, and in particular, to bring out the hitherto unexplored potential influence of ENSO Modoki on African rainfall. Further, as the rainfall variability of the tropical West Africa and Sahelian region has been a subject of critical research interest^16^,^34^, we pay a little more attention to the boreal summer monsoon. Canonical ENSO^1^,^2^,^15^ and IOD^22^,^25^ are reported to have strong influence over the tropical African region, including Sahel, which receive more than 80% of annual total rainfall during boreal summer.

## Revisiting the seasonal cycle of rainfall over Africa

Briefly, we focus on the March-April-May (MAM) and October-November-December (OND) seasons, as prominent rainfall in the central and east African region mainly occurs during these seasons^35^. June-July-August-September (JJAS) season is selected due to the propensity of the rainfall occurrence during this season in the northern, southern and West African regions^36^,^37^.

Also, January-February (JF) period is selected considering that the austral summer rains with January maximum are common mainly in north eastern parts of Southern Africa, southeast Angola and Eastern Africa south of 10 °S^2^.

**Figure 1:**
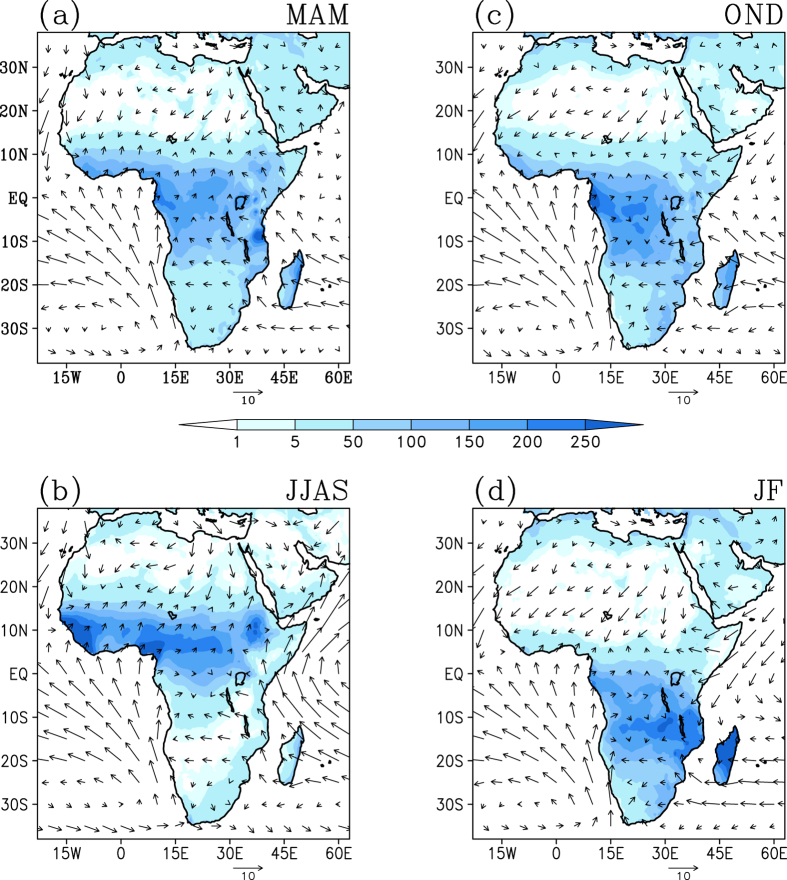
Climatology (1979–2010) of seasonal rainfall (mm/month) and surface winds (m/s). [Figure created using the COLA/GrADS software].

As shown in Fig. 1, it is apparent that the ITCZ is a major factor that governs the position and intensity of rainfall in tropical Africa. Near the equatorial region, the rainfall distribution achieves maxima during the two transition seasons. In the areas of Kenya and other regions, there is a third maximum, usually occurring in July or August^35^. The subtropical margins of both hemispheres receive maximum rainfall during the high-sun season of the respective hemisphere^35^.

The tropical west and central portions of the continent experience so called ‘long rains’ during MAM. These are associated with the seasonal migration of the solar irradiance into northern hemisphere and shift of the ITCZ to the seasonal mean position of ~13 °N (Fig. 1a). In association with further northward movement of the ITCZ to ~18 °N by the boreal summer, the rainfall band over the continent relocates between equator and 20 °N (Fig. 1b). As mentioned earlier, Sahel region receives 80% of its annual rainfall mostly during July-September, with maximum rainfall during August^16^,^34^,^36^. Rainfall in the northern subtropical regions such as Sudan is governed by the ITCZ, with maximum monthly rainfall during the months of July and August^37^. The ITCZ moves southward again in boreal fall, and is slanted southwest from 10 °N to Madagascar across the continent during OND (Fig. 1c). During this period the tropical central Africa, predominantly south of the equator, once again experiences the rains (Fig. 1c) in a year, which are referred to as the ‘short rains’. During JF, a broad region in the southern hemisphere from equator through Madagascar receives rainfall (Fig. 1d), with the maximum occurring around Madagascar. This is associated with the southward movement of the ITCZ down to 20 °S (Fig. 1d). In addition, the synoptic scale tropical temperate troughs are responsible for most of the summer rainfall over subtropical southern Africa. The ITCZ structure changes over Southern Africa/Madagascar in association with presence of the tropical temperate troughs, but remains similar to climatology over other regions^38^. We also see a secondary peak in rainfall over the south eastern parts of the continent. Incidentally, east coastal Madagascar experiences rainfall throughout the year except during JJAS. The rainfall here peaks in JF, associated with the tropical cyclone season, and often extends to March^39^. Occurrence of heavy rainfall events of more than 10 cm day^−1^ can be seen during January and February.

## Teleconnections with tropical Indo-Pacific drivers

**Figure 2:**
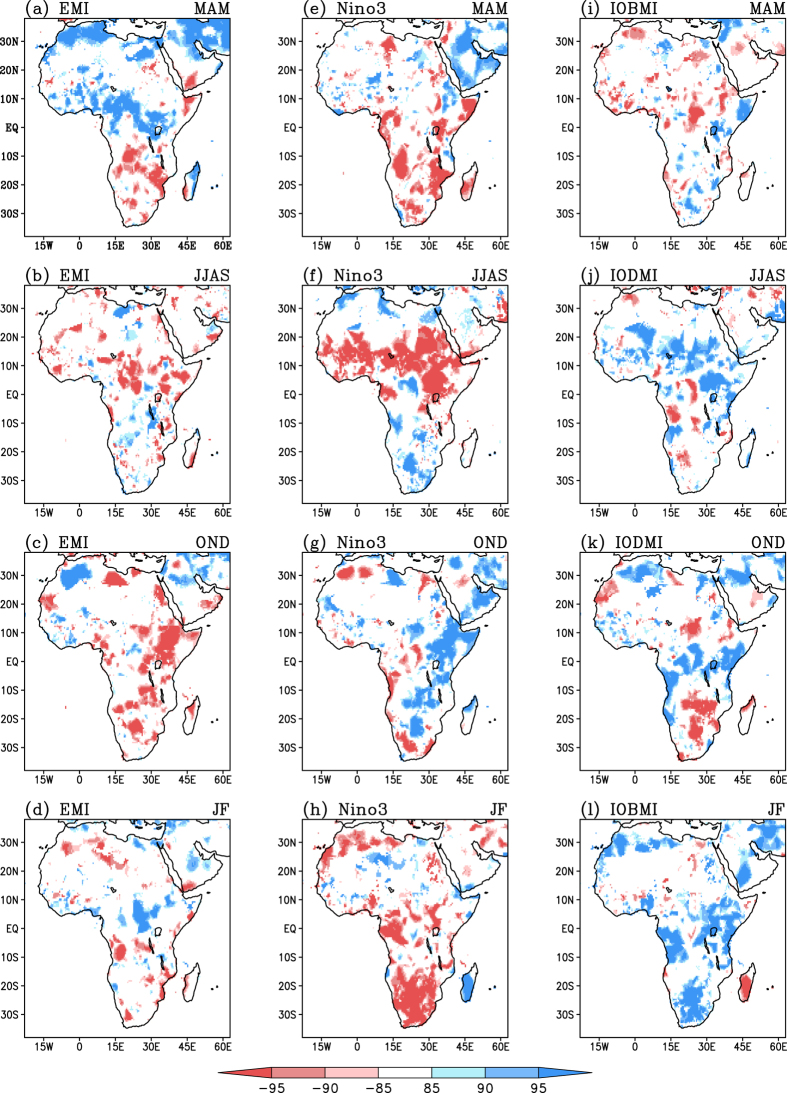
Partial correlations between CRU seasonal rainfall and SST indices. (**a**–**d**) with EMI, (**e**–**h**) with ENSO index, (**i**,**l**) with IOBMI, (**j**,**k**) with IODMI for the seasons MAM (**a**,**e**,**i**), JJAS (**b**,**f**,**j**), OND (**c**,**g**,**k**) and JF (**d**,**h**,**l**). Correlations significant at 85%, 90% and 95% confidence level, based on Student’s t-test are shown. [Figure created using the COLA/GrADS software].

The relative impacts of Indo-Pacific drivers on the African seasonal rainfall during various seasons are explored using the partial correlation analysis between the local rainfall and SST indices (Fig. 2). Figure 2a,d (Fig. 2e,h) exhibit the impact of various phases of ENSO Modoki (canonical ENSO) evolution on the seasonal rainfall. The significant impact of the IOD (IOBM) events on the African climate during JJAS and OND seasons (MAM and JF) can be discerned from Fig. 2j,k (Fig. 2i,l). The results obtained using CRU rainfall dataset (Fig. 2) is reconfirmed using GPCP (Fig. A2) satellite estimates. A composite analysis also, in general, confirms the result (Fig. A3).

### March-May

During the ‘long rain’ season, the El Niño Modoki (Fig. 2a, A2a) events have impacts distinctly different from those of canonical El Niño (Fig. 2e, A2e) and IOBM (Fig. 2i, A2i). Specifically, the El Niño Modoki events induce significant enhancement in rainfall in the tropical regions like western Kenya, Uganda, Rwanda, Central African Republic, Nigeria, Guinea, and also in north Madagascar. In contrast, significant reduction in rainfall is seen over the southern parts of Africa, especially over the regions of Angola, Namibia, Democratic Republic of the Congo, Mozambique, Zambia, regions of Botswana, and South Africa (Fig. 2a, A2a). Thus, El Niño Modoki events are in general associated with an anomalous enhancement of rainfall primarily between 5 °S to 15 °N and a reduction to the south of 5 °S. The canonical El Niño also results in significant reduction of rainfall in most of the southern hemispheric Africa (Fig. 2e, A2e). However, the basin-wide warming in the Indian Ocean is associated with significant reduction (enhancement) of rainfall over northern tropics (eastern and southern Africa) (Fig. 2i, A2i).

### June-September

Associated with the occurrence of El Niño Modoki events, a significant below-average rainfall during the boreal summer season occurs mainly over northern latitudes of Africa, over central and east African regions especially Ethiopia, parts of Kenya, Uganda, Chad, Central African Republic, and Sahel (Fig. 2b, A2b). Canonical El Niño events introduce significant below average rainfall in the northern tropics (Fig. 2f, A2f), in agreement with earlier studies^1^,^2^,^15^, more prominent as compared to those of the Modoki events. Further, canonical El Niño events induce an anomalous enhancement of rainfall over southern regions. On the contrary, significant positive correlations between the rainfall and IODMI (Fig. 2j, A2f) can be observed over northern latitudes, especially over the Sahel, Sudan and the equatorial region, in agreement with earlier studies^22^,^25^,^29^. The IOD events apparently have impacts opposite to those of the ENSO flavours in the northern tropics.

### October-December

During the ‘short rain’ season, several broad regions of eastern and southern African regions like Sudan, Ethiopia, Uganda, Kenya, north eastern regions of Dem. Rep. of the Congo, exhibit significant below-average rainfall in association with El Niño Modoki events (Fig. 2c, A2c). In contrast, significant increase in rainfall is seen in eastern Africa in association with canonical El Niño (Fig. 2g, A2g) and positive IOD events (Fig. 2k, A2k). This indicates, strictly in a linear sense, that a positive IOD event reduces the contrasting influence from a co-occurring El Niño Modoki in the eastern parts of the continent, while it amplifies the in-phase impact from a co-occurring canonical El Niño. The significant positive correlations observed between the east African rainfall and IOD events during OND season (Fig. 2k, A2k) are in general agreement with the earlier studies^21^,^22^, which show that the east African ‘short rains’ are predominantly driven by the warm SST anomalies in the western equatorial Indian Ocean. Interestingly, the eastern cold pole of the tropical IOD is of lesser relevance for the ‘short rains’^21^, which we have ascertained through a simple partial correlation analysis (Fig. A4). In tropical central Africa, predominantly south of the equator, above normal rainfall activity can occur in association with El Niño Modoki (Fig. A2c) and positive IOD (Fig. 2k, A2k) events; whereas, canonical El Niño events induce an opposite effect, i.e. a below normal rainfall activity (Fig. 2g, A2g). Therefore, the impact of any co-occurring positive IOD and canonical El Niño (El Niño Modoki) on central (east) African rainfall depends on the relative strength of these events. On the contrary, the co-occurring positive IOD and canonical El Niño (El Niño Modoki) will enhance the east (central) African rainfall.

### January-February

A reduction in rainfall activity in the southern latitudes of Africa, especially, the east African coastal region (between 15 °S to 30 °S) can be observed during the JF season in association with the El Niño Modoki events (Fig. 2d, A2d), similar to MAM season. Further, the canonical El Niño events are associated with significant reduction in rainfall over equatorial and southern regions of Africa during these months, just as during the MAM season. Whereas, Madagascar experiences significant enhancement of rainfall in association with the canonical El Niño events during the JF months, in contrast with the anomalous reduction of rainfall it experiences during the MAM season. Indian Ocean Basin-wide warming tends to produce above normal rainfall over most of the tropical and southern parts of the continent but a below normal rainfall over Madagascar. These signatures are opposite to that of the canonical El Niño events.

## Potential mechanisms behind the ENSO Modoki teleconnections to seasonal rainfall

**Figure 3:**
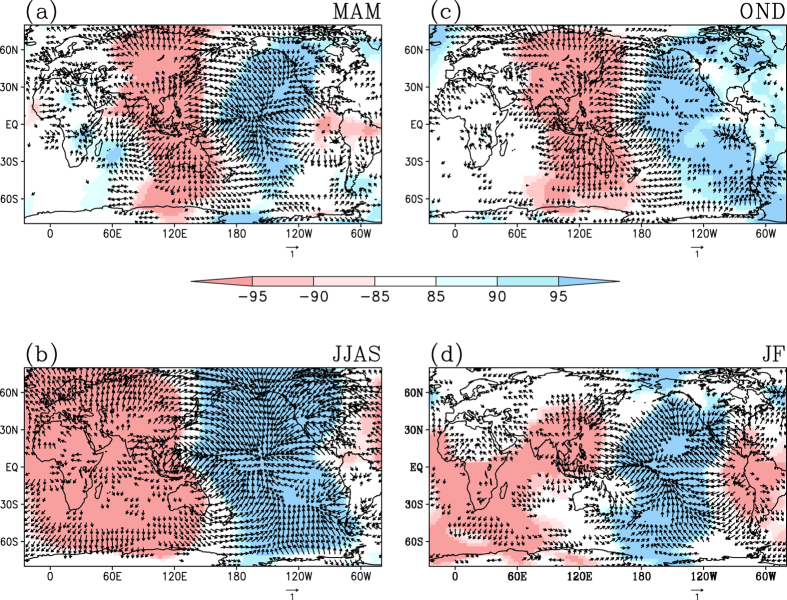
Partial correlations between EMI and velocity potential at 850 hPa (shadings). Correlations significant at 85%, 90% and 95% confidence level based on Student’s t-test are provided for different seasons (**a**) MAM, (**b**) JJAS, (**c**) OND and (**d**) JF. Partial correlations between EMI and 850 hPa divergent wind components (vectors), significant at 95% confidence level is also given for each season. [Figure created using the COLA/GrADS software].

**Figure 4:**
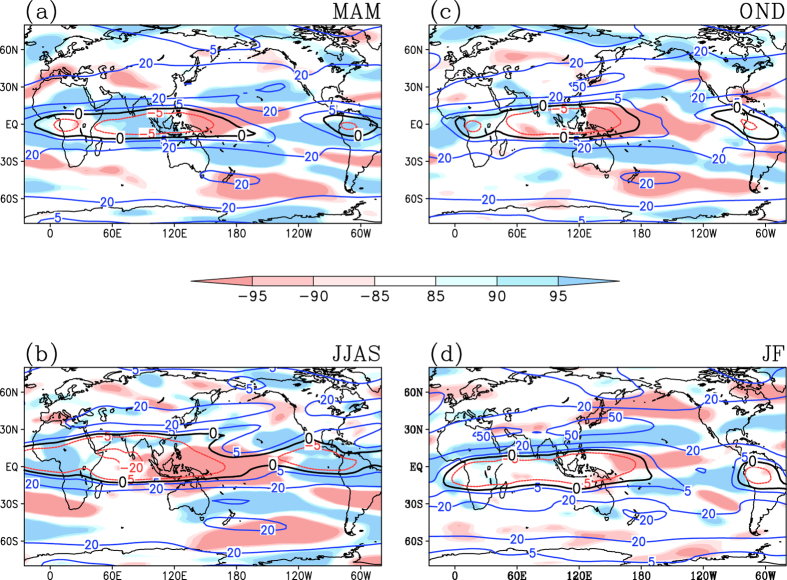
Partial correlations between EMI and zonal wind at 200 hPa, for the seasons (**a**) MAM, (**b**) JJAS, (**c**) OND and (**d**) JF. Correlations significant at 85%, 90% and 95% confidence level based on Student’s t-test are shown. Blue and red contours represent climatological westerly and easterly zonal winds (m/s) respectively. Climatological zonal winds with magnitude zero m/s is displayed in black contour. A positive correlation between westerly (easterly) winds and EMI has to be interpreted as strengthening of westerly jet (weakening of easterly jet) in association with increase in El Niño Modoki. Whereas a negative correlation between westerly (easterly) winds and EMI implies weakening of westerly jet (strengthening of easterly jet) in association with increase in El Niño Modoki. [Figure created using the COLA/GrADS software].

The ENSO Modoki events influence the tropical and subtropical African rainfall significantly by inducing anomalous changes in Walker Circulation. The partial correlations of the EMI with divergent wind at 850 hPa and those with velocity potential at 850 hPa are presented in Fig. 3. These indicate that the enhanced rainfall activity over north Madagascar and tropical regions of Africa (Fig. 2a, A2a) in MAM season during an El Niño Modoki is associated with an anomalous zone of large scale convergence over southwest Indian Ocean and the east coast of Africa (Fig. 3a). The northwest Africa receives a significant part of its spring rainfall through synoptic systems associated with baroclinic instability^40^. Interestingly, we find that El Niño Modoki events are associated with strengthening of the subtropical jet stream over the northern regions of Africa (Fig. 4a) during MAM season. This suggests that the anomalous increase in rainfall during the El Niño Modoki (Fig. 2a) is manifested through strengthening of interactions between the mean flow and synoptic activity.

During the boreal summer, the occurrence of El Niño Modoki are associated with anomalous subsiding limb of the Walker Circulation in the tropical Africa, indicated by significant large-scale low level subsidence (Fig. 3b) and reduction in rainfall in northern tropical Africa (Fig. 2b, A2b). In addition, significant weakening of the tropical easterly jet stream (TEJ) is seen (Fig. 4b) in association with the El Niño Modoki events. However, the TEJ and convection are reported to be linked over West Africa; east of 20 °E the location of TEJ may promote convection while west of 10 °E it is the convection that controls the TEJ^41^.

Further, an anomalous enhancement of rainfall along the tropical central African region (Fig. A2c) and a reduction in rainfall along the southern and eastern part of Africa are associated with the El Niño Modoki events during the OND season (Fig. 2c, A2c), and apparently due to an anomalous convergence zone that flanks the off-equatorial southwest Africa (Fig. 3c). During the JF season, El Niño Modoki events apparently induce a broad anomalous divergence in the southern hemispheric regions of Africa (Fig. 3d), which in turn induces rainfall deficits over east and southern regions of Africa in the southern hemisphere, and enhanced rainfall over tropical northern latitudes (Fig. 2d, A2d). The El Niño Modoki events are apparently associated with an anomalous weakening of the subtropical jet stream over northern regions of Africa during OND (Fig. 4c); in contrast, subtropical jet stream during JF season anomalously intensifies in association with the El Niño Modoki events (Fig. 4d).

## Impact of Indo-Pacific drivers on boreal summer Tropical African and Sahelian rainfall

Tropical west and Sahelian regions are highly sensitive to the SST variability in all tropical basins, both remote (Pacific) and neighbouring (Atlantic and Indian). However, much of the interannual variations can be accounted for by the remote effect of the tropical Pacific SSTs only^42^. El Niño type warming and weakened large-scale zonal gradient of SSTs from west Pacific to the east Indian Ocean increases the likelihood of Sahel drought. Their Sahelian impact is reinforced when both are present^43^.

**Figure 5:**
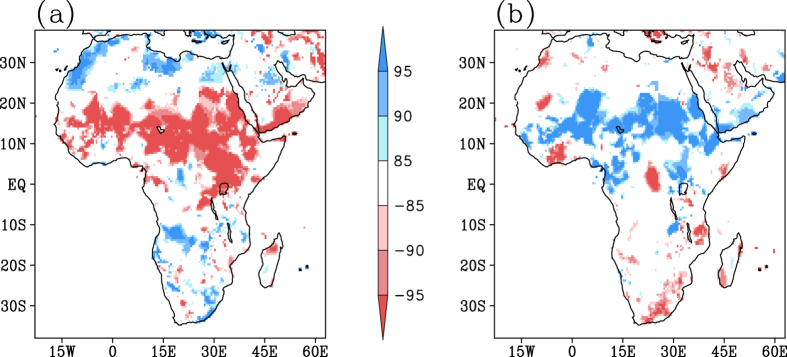
Correlation of zonal winds averaged over the jet core regions of (**a**) TEJ [15 °W-45 °E; Eq-10 °N] and (**b**) AEJ [15 °W-40 °E; 10 °N-20 °N] with JJAS rainfall. Correlations significant at 85%, 90% and 95% confidence level based on Student’s t-test are shown. A positive correlation implies an enhancement (reduction) of rainfall in association with weakening (strengthening) of TEJ/AEJ. Whereas a negative correlation implies a reduction (enhancement) of rainfall with weakening (strengthening) of TEJ/AEJ. [Figure created using the COLA/GrADS software].

To describe the possible mechanisms of the impacts of the tropical Indo-Pacific drivers, it is important to understand the influence of the drivers on African easterly jet (AEJ) and TEJ, in addition to studying the relevant Walker circulation changes with the variability of these drivers. The boreal summer rainfall over Sahel exhibits significant anomalous reduction (enhancement) in association with weakening (strengthening) of TEJ and strengthening (weakening) of AEJ^44^ (Fig. 5). During the wet years, a northward displacement of AEJ and an associated enhancement in both the horizontal and vertical wind shear related to the climatological conditions is observed over the Sahel, and is associated with baroclinic instability^44^. South of the Sahel, on the other hand, both the location and intensity of the AEJ appear to play an important role, and the dominant instability mechanism appears to be barotropic^44^. A possible offshoot of the increase in the strengths of AEJ, especially towards southern latitudes can shift the zone of unstable squall inducing waves slightly southwards and causes an anomalous deficit in rainfall in West Africa, north of latitude 12 °N^45^.

**Figure 6:**
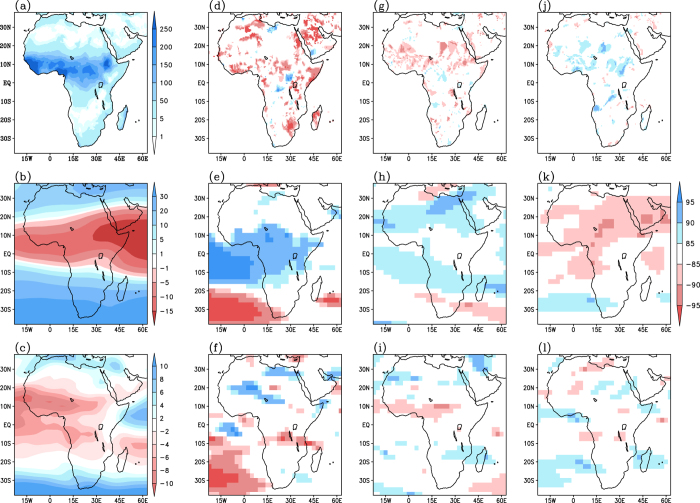
First column (**a,b,c**) shows, respectively, climatological rainfall (mm/month), 200 hPa zonal winds (m/s) and 700 hPa zonal winds (m/s) during JJAS season for the period 1979–2010. The corresponding composited anomalies for El Niño Modoki, obtained from composite analysis, are shown in second column (**d–f**). The corresponding anomalies for El Niño and positive IOD years are shown in third (**g–i**) and fourth columns (**j–l**), respectively. Significant values at 85%, 90% and 95% confidence level based on Student’s t-test are shown in shadings. [Figure created using the COLA/GrADS software].

To get more insight into the Modoki impacts, we carry out a composite analyses of various anomalous fields of rainfall, zonal winds at 200 hPa and 700 hPa (where the TEJ and AEJ respectively have their maximum intensity) over seven El Niño Modoki summers (Fig. 6d–f). As a reference, we also present the observed climatology in Fig. 6a–c. An anomalous reduction in rainfall over tropical northern latitudes can be seen in Fig. 6d, and also from partial correlation analysis (Fig. 2b, A2b). However, there is some discrepancy between the results from the composites and partial correlations, especially over the region encompassing 28 °E-32 °E; 5 °S-12 °S near Lake Malawi. This may be potentially due to some non-linearity in the impacts between the positive and negative events, which cannot be captured by the linear partial correlation analysis. In association with the occurrence of El Niño Modoki events, we see significant anomalous weakening of TEJ (Fig. 6e). Also seen is the anomalous strengthening of AEJ along 10 °S and between Equator and 10 °N, suggesting a possible southward shift in the respective axis of AEJ (Fig. 6f). Such changes in the jet streams are expected to anomalously reduce the rainfall over Sahel region. Indeed this apparently happens during the El Niño Modoki events.

A similar composite analysis over the canonical El Niño events also show an anomalous weakening of TEJ (Fig. 6h), consistent with the earlier study^46^, a strengthening and southward shift of AEJ (Fig. 6i), and a reduction in rainfall over the tropical northern Africa (Fig. 2f, A2f and 6g). Further, the Sahel region experiences significantly drier conditions in canonical El Niño summers as compared to the El Niño Modoki (Fig. A5a), in agreement with Figs 2b (A2b) and 2f (A2f). Also, the anomalous changes in the TEJ and AEJ are significantly stronger in magnitude during the canonical El Niño (Fig. A5b-c). This suggests that the qualitatively similar impact of the two El Niño types on the African rainfall arises from a similar mechanism. A mechanism for Pacific teleconnection to Sahel also may involve anomalous stationary waves, with communication occurring in both the eastward and westward directions. Strong anomalous weakening of TEJ between 90 °W and 90 °E associated with El Niño events has been reported as a Kelvin wave response^46^. During the El Niño years, a Kelvin wave emanates across the tropical Atlantic from the tropical east Pacific convective heating anomalies. Further, an equatorial Rossby wave appears over Indian Ocean in response to the anomalous west Pacific-Indian Ocean SST gradient. These waves interact over Africa and induce an anomalous large scale subsidence over Sahel, thus anomalously reducing seasonal rainfall totals^43^.

A composite analysis over the positive IOD events, reveals an anomalously positive rainfall signal over the tropical northern Africa (Figs 2j, A2j and 6j) in association with anomalous strengthening of TEJ (Fig. 6k) and weakening of AEJ (Fig. 6l). This suggests that the IOD events also could influence the African rainfall through modulating the intensity of jet streams. This mechanism is different from the well-known mechanisms such as the anomalous low level convergence patterns induced over east Africa as a direct response to the anomalous warming in the tropical west Indian Ocean during a positive IOD event^22^,^25^,^26^.

**Figure 7:**
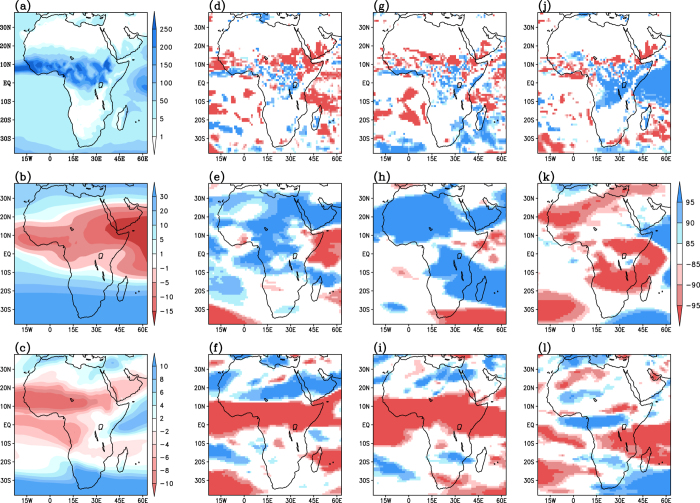
First column (**a–c**) shows, respectively, simulated rainfall (mm/month), 200 hPa zonal winds (m/s) and 700 hPa zonal winds (m/s) during JJAS season from the control experiment. The simulated differences for the El Niño Modoki experiment (obtained by subtracting the control simulation from the El Niño Modoki simulation) are shown in second column (**d–f** represent rainfall, 200 hPa and 700 hPa zonal winds respectively). The corresponding differences for the El Niño experiment and positive IOD experiment are shown in third (**g–i**) and fourth columns (**j–l**) respectively. Significant values at 85%, 90% and 95% confidence level based on Student’s t-test are shown in shadings. [Figure created using the COLA/GrADS software].

To confirm that such an attributed change in zonal winds during the boreal summer is indeed due to the forcing from Indo-Pacific events, we perform the following several sensitivity experiments using the LMDZ (Laboratoire de Meteorologie Dynamique and Z stands for Zoom) Atmospheric General Circulation Model (AGCM), (a) a control experiment with climatological SST, and experiments in which the AGCM is forced with SSTs representative of a typical (b) El Niño Modoki, (c) canonical El Niño and (d) positive IOD. Before analysing the results, we ascertain the ability of the LMDZ model in simulating the climatological rainfall and zonal winds over Africa. A comparison between the observations (Fig. 6a–c) and model-simulations obtained from the control run (Fig. 7a–c), demonstrates that the model, in general, simulates a reasonable spatial distribution of the JJAS rainfall and zonal winds over Africa. Intensity and general position of core axis of the TEJ and AEJ are also in good agreement with the observations.

The simulated seasonal mean differences in climate variables between the El Niño Modoki experiment and the control experiment (Fig. 7d–f) can be interpreted as the response to the imposed El Niño Modoki type of SSTA pattern. The observed anomalously suppressed rainfall activity in the latitude belt north of 8 °N associated with the El Niño Modoki events (Figs 2b, A2b and 6d) is well simulated by the model (Fig. 7d). However, the anomalous enhancement of rainfall simulated over equatorial region south of the dry belt is not in conjunction with observations, which could be due to the inability of the model in reasonably simulating the observed precipitation response associated with Pacific warm events. Importantly, the model results clearly show an anomalous weakening of TEJ over Africa (Fig. 7e) and a strengthening of AEJ between Equator and 10 °N (Fig. 7f), i.e. a southward shift of AEJ, in agreement with the observational findings (Fig. 6e,f), supporting our conjecture that ENSO Modoki events impact the strength and location of TEJ and AEJ and thereby influence the Sahelian and tropical African rainfall.

The simulated difference between the El Niño experiment and control experiment also show a weakening of TEJ (Fig. 7h), strengthening of AEJ between Equator and 10 °N (Fig. 7i) and an associated anomalous suppression of rainfall activity in the northern tropical latitude (Fig. 7g), consistent with the observations (Fig. 6g–i). Our model results (Fig. A5d-f) confirm the significantly stronger impacts of canonical El Niño on Sahel as compared to those from the El Niño Modoki as seen in observations (Fig. A5a-c). Just as in the Modoki experiment, an unrealistic simulation of an above average rainfall activity is seen over equatorial region in the El Niño.

Difference in the rainfall between positive IOD and control experiment (Fig. 7j) confirms the observed anomalous increase in east African rainfall (Figs 2j, A2j and 6j) during positive IOD events. However, the model does not reproduce anomalous enhancement in rainfall north of 10 °N and the reduction in rainfall over western Africa. Notwithstanding the above limitation, the simulations confirm the positive IOD-induced anomalous strengthening of TEJ (Fig. 7k) and weakening of AEJ (Fig. 7l), as evident from observations (Fig. 6k,l), the conditions favourable for an anomalous enhancement in rainfall over the tropical northern Africa. However, the anomalous weakening of AEJ in the model does not extend westward past 5 °W whereas the observation suggest a broader weakening pattern extending past 20 °W.

## Discussion

The study suggests that the ENSO Modoki events significantly, and distinctly, affect the seasonal rainfall Africa. During JJAS season, both the canonical ENSO and ENSO Modoki exhibit similar signatures on seasonal rainfall variability in Africa. On the other hand, the impacts are opposite in east African region during the OND season. We also show that a co-occurrence of an ENSO flavour with the tropical Indian Ocean drivers, namely, the IOD or the IOBM results in interference of signals, leading to either enhancement or reduction in the impact of the Pacific drivers on African rainfall.

During the MAM season, the El Niño Modoki is associated with anomalous surplus rainfall in the northern tropical Africa. However, in the southern parts of the Africa, either type of the El Niño anomalously reduces the rainfall. During the JJAS season, the El Niño Modoki events, just as the canonical El Niño events, cause an anomalous reduction of rainfall in the tropical northern latitudes of Africa, however with relatively weaker amplitude as compared to the canonical flavour. On the contrary, an anomalously enhanced rainfall signal is noted during a positive IOD event during the season, especially in the Sahel and tropical Africa. Hence, a co-occurrence of a positive IOD event, in a linear sense, could reduce the impact from the tropical Pacific drivers.

The El Niño Modoki and canonical El Niño are associated with opposite impacts on the east African rainfall during the OND season, with below (above) average rainfall during the El Niño Modoki (canonical El Niño).The positive IOD events are associated with above normal rainfall activity over east Africa. Thus a co-occurrence of an El Niño Modoki and positive IOD events can be expected to reduce each other’s impact on the east African rainfall. In contrast, the co-occurrence of canonical El Niño and positive IOD events will result in a mutual reinforcement of the individual impacts, and thereby result in a relatively strong anomalous enhancement in rainfall in east Africa. On the contrary, in central Africa, we find a destructive interference of individual impacts between the canonical El Niño and positive IOD events. From the above discussion, we can, in a linear sense, visualize the combined impacts of the co-occurring tropical Indo-Pacific drivers of various phases.

During the JF season, both the Pacific drivers have similar impact on rainfall over southern parts of Africa. Below normal rainfall occurs in association with El Niño Modoki and canonical El Niño, while above normal rainfall activities occur in association with Indian Ocean Basin-wide warming. However, in Madagascar, below (above) average rainfall activity is associated with El Niño Modoki (canonical El Niño), whereas Indian Ocean Basin-wide warming could result in below normal rainfall activity.

We find that the El Niño Modoki events, just as the other Indo-Pacific drivers, influence the seasonal rainfall variability in the tropical Africa through anomalous anomalous baroclinic changes such as a displaced Walker circulation. We also find from our observational analysis and AGCM experiments that, in addition to this mechanism, all the tropical Indo-Pacific drivers influence the boreal summer rainfall in tropical Africa by modulating the position and intensity of the AEJ and TEJ, which play an important role in governing the instability mechanisms for the rain producing systems in northern parts of Africa. Specifically, the El Niño Modoki and canonical El Niño are associated with an anomalous strengthening and a southward shift of AEJ and weakening of TEJ, thereby an anomalous reduction of rainfall in northern tropical Africa including Sahel region. Our results suggest that the positive IOD events, in contrast to the El Niño flavours, anomalously weaken (strengthen) the AEJ (TEJ), and enhance the rainfall in northern tropical African region.

The study is subject to somewhat subjective definitions of seasons such as JF, which are of course, based on earlier studies. Further, given the data quality issues, some noisy correlations, despite being statistically significant, may be irrelevant. Therefore, further verification of these results using several other AGCMs and GCMs will be helpful. Further, while an examination of asymmetric response of the rainfall in different African regions to the opposite phases of the tropical Indo-Pacific drivers will be also an interesting issue, it is beyond the scope of the current work owing to our linear analysis methodology and limited number of the AGCM experiments. Notwithstanding these issues, the work provides, for the first-time, a comprehensive appraisal of the potential impacts of the ENSO Modoki on the African climate with emphasis on equatorial east Africa and the West African monsoon region. Thus, the work provides background information for applications such as seasonal prediction.

## Methods

### Data

We use the Climate Research Unit (CRU) time series (TS) version 3.20 of high resolution monthly precipitation data^47^, circulation datasets from the National Center for Environmental Prediction/National Center for Atmospheric Research (NCEP/NCAR) reanalysis product^48^, and the Hadley centre sea ice and sea surface temperatures [HadISST]^49^ developed at Met Office, Hadley Centre for Climate Research, all for the period from 1979 to 2010. We also use available satellite rainfall estimates from the version-2 Global Precipitation Climatology Project (GPCP)^50^ to reconfirm the results obtained from the analysis of CRU data. All our analysis has been carried out using both detrended and raw data. The results obtained are found to be robust to the trend analysis. Therefore, only the analysis carried out using the detrended data is included in the paper.

To represent the canonical ENSO, ENSO Modoki, IOD and Indian Ocean Basin Mode events, we use the NINO3 index, ENSO Modoki Index (EMI), Indian Ocean Dipole Mode Index (IODMI) and the Indian Ocean Basin Mode Index (IOBMI), respectively. The NINO3 index is defined as the area-averaged sea surface temperature anomalies (SSTA) over the region bounded by 5 °N-5 °S and 150 °W-90 °W. The EMI^23^ is defined as, EMI = [SSTA]_C_ − 0.5 [SSTA]_E_ − 0.5 [SSTA]_W_, where the square bracket with a subscript represents the area mean SSTA, averaged over one of the three regions specified as the central (C: 165 °E-140 °W, 10 °S-10 °N), eastern (E: 110°-70 °W, 15 °S-5 °N), and western (W: 125°-145 °E, 10 °S-20 °N) regions of the tropical Pacific. The IODMI^22^ is defined as the SSTA difference between the western (50 °E-70 °E; 10 °S-10 °N) and southeastern (90 °E-110 °E; 10 °S-Equator) regions of the tropical Indian Ocean. The IOBMI^51^,^52^ is obtained as the SST anomaly area-averaged over the Indian Ocean region bounded by 20 °S-20 °N and 40 °E-110 °E.

Importantly, while the IOD events occur during boreal summer and fall, the IOBM events are mainly prominent during the boreal winter and spring. We therefore consider only one of these two as the main driver from the tropical Indian Ocean depending on the season under consideration. It is to be noted, that IOD are largely independent of ENSO^53^, particularly through the boreal summer. While the correlation between the respective indices is indeed significant during the boreal fall, many IOD events such as those during 1961, 1963, 1967, etc. occurred without a concurrent El Niño. The IOBM events on the other hand are strongly associated with ENSO^18^,^19^,^20^,^21^. However, the relation is not simultaneous; the IOBM event, for example, peaks with 4–5 month lag to that of an ENSO event^54^. Further, the ENSO-associated signals in the atmospheric and ocean circulation also change across the months and seasons, at least gradually. Therefore, notwithstanding such a connection with tropical Pacific, the warming in the tropical Indian Ocean can act as a heat source in its own, and through a Matsuno-Gill response, induce a teleconnection that is distinct from the impacts from tropical Pacific^55^,^56^,^57^. Based on all these, we treat the IOBM and ENSO as different drivers of climate variability in Africa during boreal winter and spring seasons.

In this study, to isolate the individual impacts of the tropical Indo-Pacific drivers on the African rainfall variability, we employ the well-accepted partial correlation method.^23^,^25^,^53^. Partial correlation between the dependent variable and an independent predictor is obtained by linearly removing the effect of other independent variables in question. Here, the time series of gridded rainfall, and the SST indices namely EMI, NINO3, IOD/IOBM, for different seasons of the period 1979–2010, have been used to compute partial correlations. For example, the partial correlation between African seasonal rainfall and EMI is obtained after removing the influence of NINO3 and IOD/IOBM (depending on the season) indices. Using such a technique is essential because the life cycle of the tropical Indo-Pacific phenomena are seasonally phase locked, and co-occur together in many years, such as during the positive IOD and El Niño during the boreal summer and autumn seasons of the year 1997. In such situations, partial correlation provides a more realistic estimate of the individual impact of the drivers as compared to linear correlations, at least from a linear perspective^23^,^25^,^53^. Correlation coefficient of OND rainfall and IODMI, both linear and partial (on removal of influence from western and eastern pole of IOD respectively), has been carried out to identify the relative importance of the western and eastern pole.

We also implement a composite analysis technique for an understanding of the impact of the tropical Indo-Pacific drivers on African rainfall during boreal summer season. Seven El Niño Modoki years (1986, 1990, 1991, 1992, 1994, 2002 and 2004), four El Niño years (1982, 1983, 1987 and 1997), four positive IOD years namely 1999, 2003, 2007 and 2008, and three positive IOBM years (1988, 1998 and 2005) were selected for the composite analysis. Out of the four positive IOD events selected, two of them (1999 and 2007) occurred during the development of La Niña events. The SST indices and basis for the selection of years are narrated in our earlier papers^23^,^31^.

To understand the influence of TEJ and AEJ on boreal summer rainfall of Africa, correlations between a zonal wind index for TEJ and AEJ with the JJAS rainfall have been performed. An index for the TEJ variability is obtained by area-averaging of Zonal winds at 200 hPa in the TEJ core region, bounded by[15 °W-45 °E; Eq-10 °N]. The corresponding index for the AEJ is obtained by area-averaging of the 700 hPa zonal winds over the region bounded by [15 °W-40 °E; 10 °N-20 °N. The statistical significance of the results obtained from the linear and partial correlation, and composite analysis are assessed by using a one tailed Student’s t-test. Results significant at 85%, 90% and 95% confidence level are displayed in the figures.

### Model

The LMDZ AGCM^58^ has been developed at Laboratoire de Meteorologie Dynamique (LMD), France. It has a horizontal resolution of 1.5° × 1.25° and 39 vertical levels. This model is used to perform various sensitivity experiments to explore the potential mechanism behind the impacts of the tropical Indo-Pacific drivers *during boreal summer season*. Four sets of experiments were carried out, each with ten ensemble members with perturbed initial conditions of 1 January, and integrated for 9 months. In the first experiment, henceforth referred to as the control experiment, the AGCM is forced with monthly varying climatological (1979–2010) SST, derived from the HADISST, as the lower boundary conditions. In the second experiment, what can be referred to as the El Niño Modoki experiment, the AGCM is forced with El Niño Modoki type of SST, obtained by imposing monthly June to September El Niño Modoki type of SST anomalies on the climatological SSTs only in the tropical Pacific region (110 °E–90 °W, 15 °S–15 °N), following previous studies^59^. These monthly anomalies are computed in a two-step process: for each month, (i) a composite value of the EMI is obtained by averaging it over pure strong El Niño Modoki years of 1994, 2002 and 2004; and (ii) this composite EMI is multiplied with the correlation coefficients between the SSTA and EMI at every grid point. Similarly, the third (El Niño) and fourth (positive IOD) experiments are carried out, using monthly SST anomalies obtained from the above two-step process. The years used to design the SST anomalies for the canonical El Niño are 1982, 1987 and 1997, and those for the positive IOD are 2003, 2007 and 2008. Following earlier studies^26^,^27^, in the El Niño experiments, the SST anomalies are imposed on the climatological SSTs only in the tropical Pacific (110 °E–90 °W, 15 °S–15 °N), while in the positive IOD experiment, the SST anomalies were imposed on the climatological SST only in the tropical Indian Ocean region (35 °E-115 °E, 30 °S-30 °N). Figure A6 provides the imposed SSTA boundary conditions used for forcing the sensitivity experiments for the month of September, as an example. As is the practice, the seasonal (JJAS) ensemble mean differences in climate variables between the three experiments and the control experiment can be interpreted as the responses induced by El Niño Modoki/El Niño/positive IOD events during the boreal summer.

## Additional Information

**How to cite this article**: Preethi, B. *et al.* Impacts of the ENSO Modoki and other Tropical Indo-Pacific Climate-Drivers on African Rainfall. *Sci. Rep.* **5**, 16653; doi: 10.1038/srep16653 (2015).

## Supplementary Material

Supplementary Information
