# Erratum: Impacts of the ENSO Modoki and other Tropical Indo-Pacific Climate-Drivers on African Rainfall

**DOI:** 10.1038/srep20266

**Published:** 2016-01-28

**Authors:** B. Preethi, T. P. Sabin, J. A. Adedoyin, K. Ashok

Scientific Reports 5: Article number: 1665310.1038/srep16653; published online: 11162015; updated: 01282016

This Article contains errors in the Reference list. References 35, 36 and 37 were incorrectly listed as references 37, 35 and 36 respectively. In addition, there is a typographical error in Reference 35. The correct references are listed below:

Reference 35:

Nicholson, S. E. A review of climate dynamics and climate variability in eastern Africa. *The Limnology, Climatology and Paleoclimatology of the East African Lakes* (eds. Johnson, T. C. & Odada E.) 25-56 (Gordon and Breach, 1996).

Reference 36:

Nicholson, S. E. & Palao, I. M. A re-evaluation of rainfall variability in the Sahel. Part 1. *Characteristics of rainfall fluctuations. Int. J. Climatol.* **13**, 371-389 (1993).

Reference 37:

Osman, Y. Z. & Shamseldin, A. Y. Qualitative rainfall prediction models for central and southern Sudan using El Niño-Southern Oscillation and Indian Ocean sea surface temperature indices. *Int. J. Climatol.* **22**, 1861-1878 (2002).

