# Supplementary material for: Impacts of the ENSO Modoki and other Tropical Indo-Pacific Climate-Drivers on African Rainfall
Source: Sci Rep. 2015 Nov 16;5:16653. doi: 10.1038/srep16653 (PMC4645176; doi:10.1038/srep16653)
Supplement: Supplementary Information [file srep16653-s1.pdf]

Auxiliary Material

for

Impacts of the ENSO Modoki and other Tropical Indo-Pacific Climate-Drivers on African Rainfall

B. Preethi[1], T. P. Sabin[1], J. A. Adedoyin[2], K. Ashok[13*]

[1]Indian Institute of Tropical Meteorology, Pune – 411 008, India
[2]Physics Department, University of Botswana, Gaborone, Botswana
[3]University Centre for Earth and Space Sciences, University of Hyderabad, Telangana, India
[*]Corresponding Author, Email: ashokkarumuri@uohyd.ac.in

The supporting information contain Auxiliary Figures A1, A2, A3, A4, A5 and A6

Auxiliary Figure Captions

Figure A1. Map of African continent. [Figure created using the COLA/GrADS software].

Figure A2. Partial correlations between GPCP seasonal rainfall and SST indices. (a) to (d) with EMI, (e) to (h) with ENSO index, (i) and (l) with IOBMI, (j) and (k) with IODMI for the seasons MAM (a, e, i), JJAS (b, f, j), OND (c, g, k) and JF (d, h, l). Correlations significant at 85%, 90% and 95% confidence level, based on Student's t-test are shown. (Same as Figure 2 but for GPCP dataset). [Figure created using the COLA/GrADS software].

Figure A3. Climatological seasonal mean rainfall (a) MAM, (b) JJAS, (c) OND and (d) JF. The percentage departure of rainfall from seasonal mean for El Niño Modoki (1986, 1990, 1991, 1992, 1994, 2002 and 2004), El Niño (1982, 1983, 1987 and 1997), positive IOD (1999, 2003, 2007 and 2008), and positive IOBM (1988, 1998 and 2005) composites are tabulated below for the highlighted region for respective season. [Figure created using the COLA/GrADS software].

Figure A4. (a) Linear correlation between OND rainfall and IODMI. (b) Partial correlation between OND rainfall and IODMI, on removal of the influence from IOD western pole. (c) same as (b) but on removal of influence from IOD eastern pole. Correlations significant at 85%, 90% and 95% confidence level, based on Student's t-test are shown. [Figure created using the COLA/GrADS software].

Figure A5. Difference between the mean JJAS anomalies, obtained from composite analysis, of (a) rainfall, (b) 200 hPa zonal wind and (c) 700 hPa zonal wind, associated with the canonical El Niño and that during El Niño Modokis. The corresponding difference between the canonical El Niño experiment and the El Niño Modoki experiments are shown in panels d, e and f. Significant values at 85%, 90% and 95% confidence level based on Student's t-test are shown in shadings. [Figure created using the COLA/GrADS software].

Figure A6. The imposed SSTA during September used as boundary forcing to the (a) El Niño Modoki, (b) El Niño and (c) positive IOD experiments. [Figure created using the COLA/GrADS software].

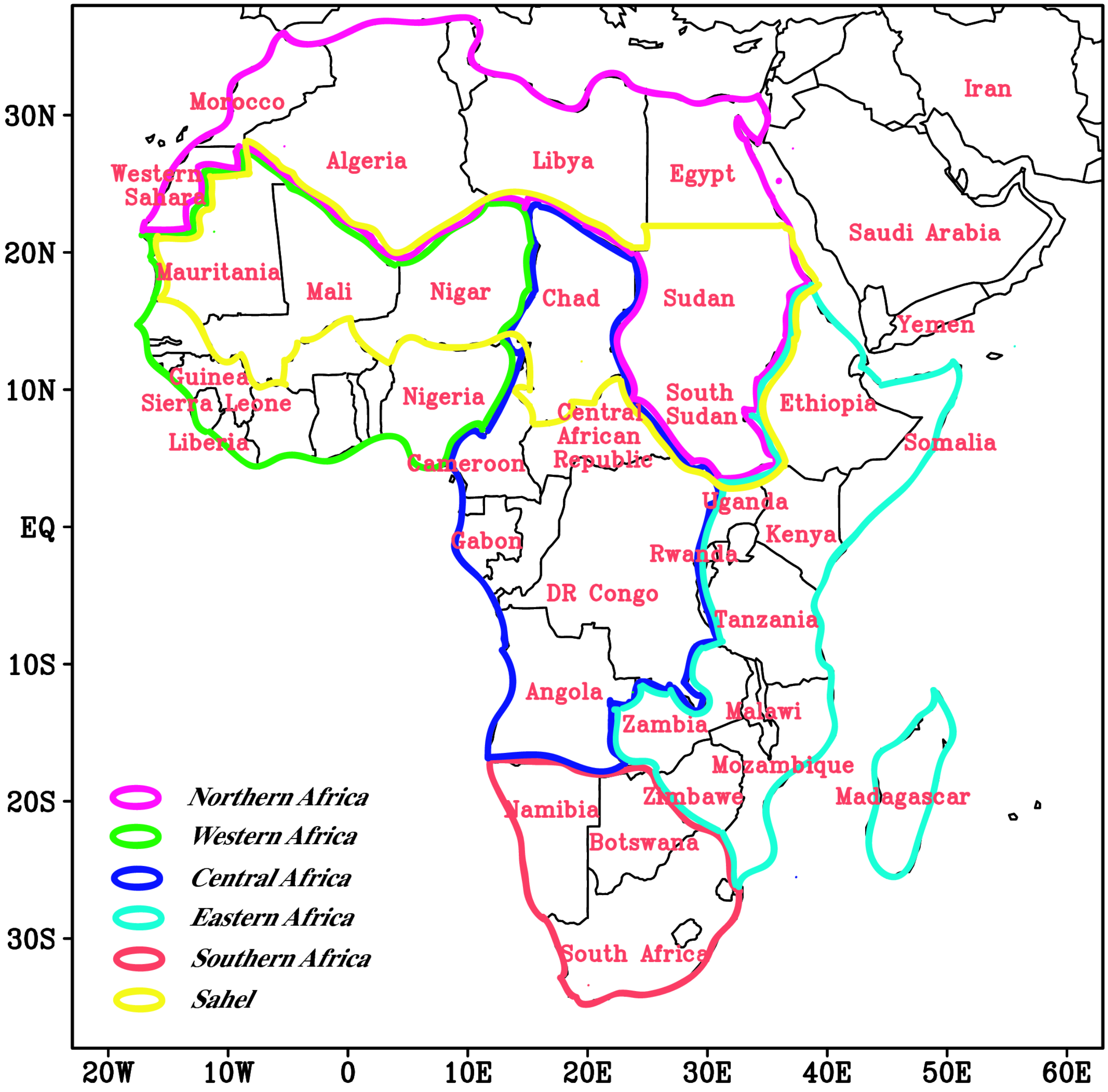

38 **Figure A1.** Map of African continent. [Figure created using the COLA/GrADS software].

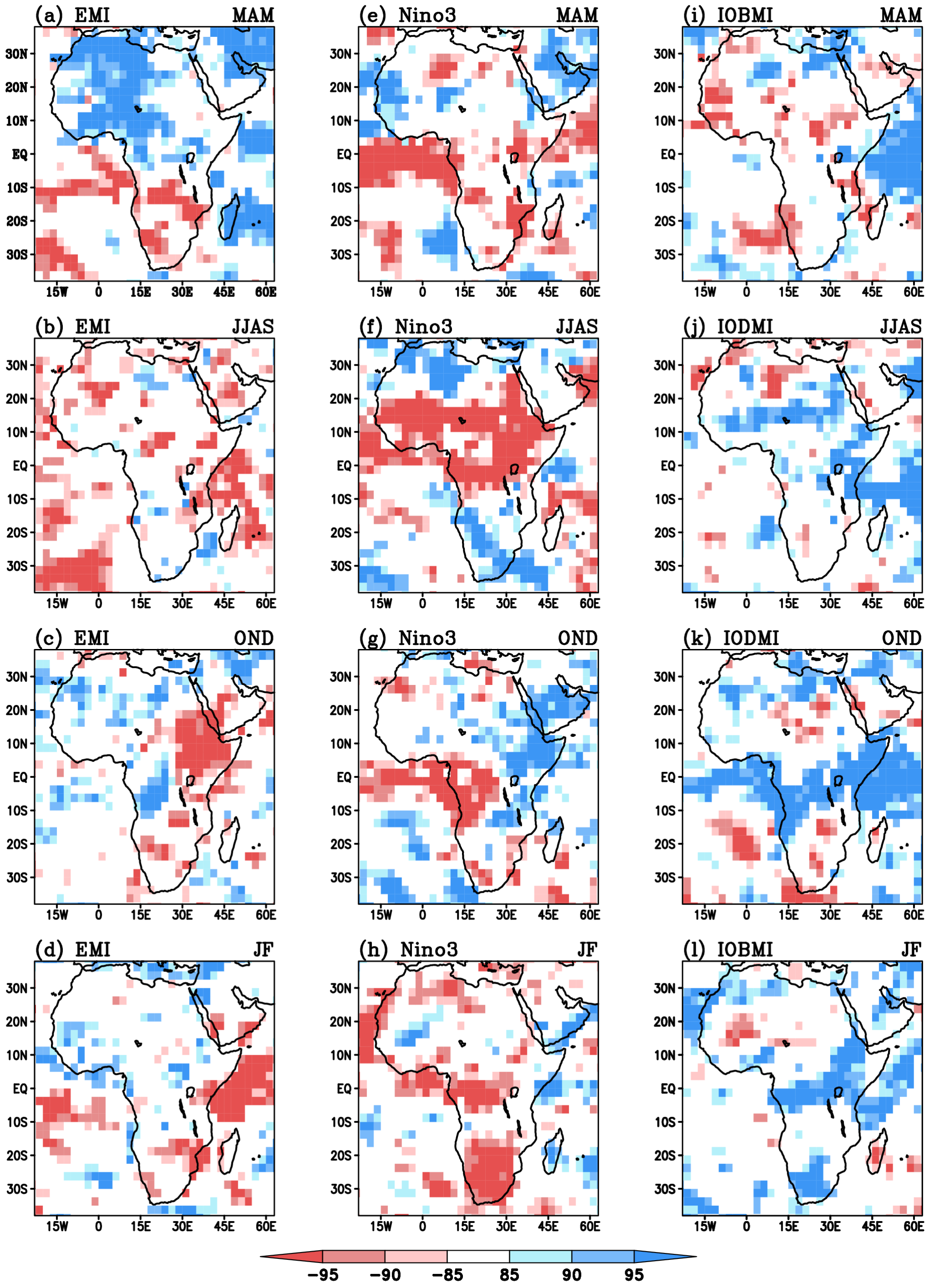

Figure A2. Partial correlations between GPCP seasonal rainfall and SST indices. (a) to (d) with EMI, (e) to (h) with ENSO index, (i) and (l) with IOBMI, (j) and (k) with IODMI for the seasons MAM (a, e, i), JJAS (b, f, j), OND (c, g, k) and JF (d, h, l). Correlations significant at 85%, 90% and 95% confidence level, based on Student's t-test are shown. (Same as Figure 2 but for GPCP dataset). [Figure created using the COLA/GrADS software].

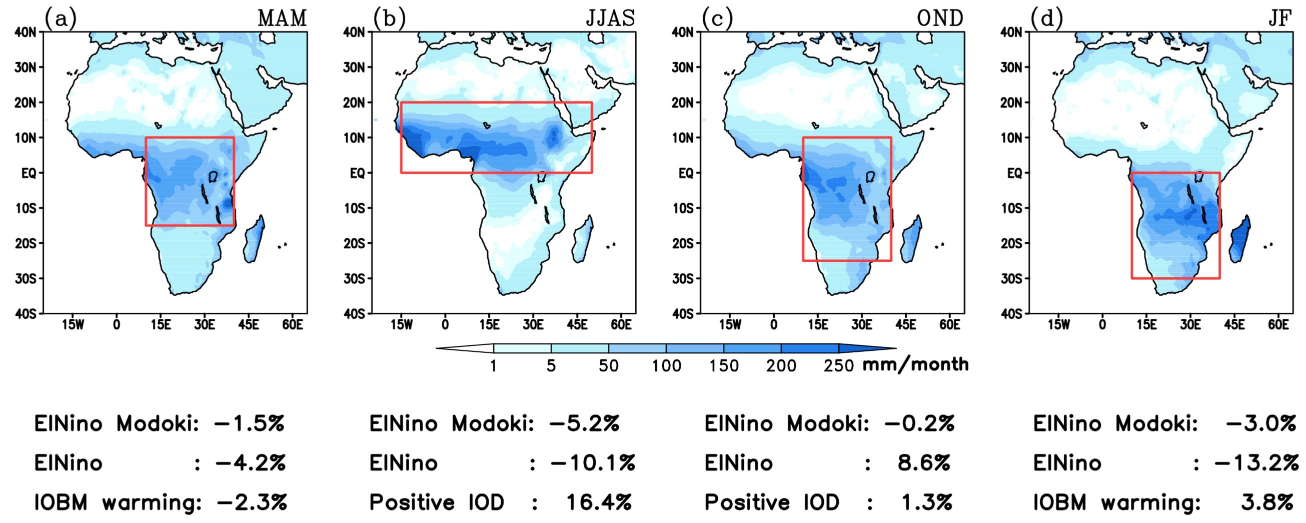

Figure A3. Climatological seasonal mean rainfall (a) MAM, (b) JJAS, (c) OND and (d) JF. The percentage departure of rainfall from seasonal mean for El Niño Modoki (1986, 1990, 1991, 1992, 1994, 2002 and 2004), El Niño (1982, 1983, 1987 and 1997), positive IOD (1999, 2003, 2007 and 2008), and positive IOBM (1988, 1998 and 2005) composites are tabulated below for the highlighted region for respective season. [Figure created using the COLA/GrADS software].

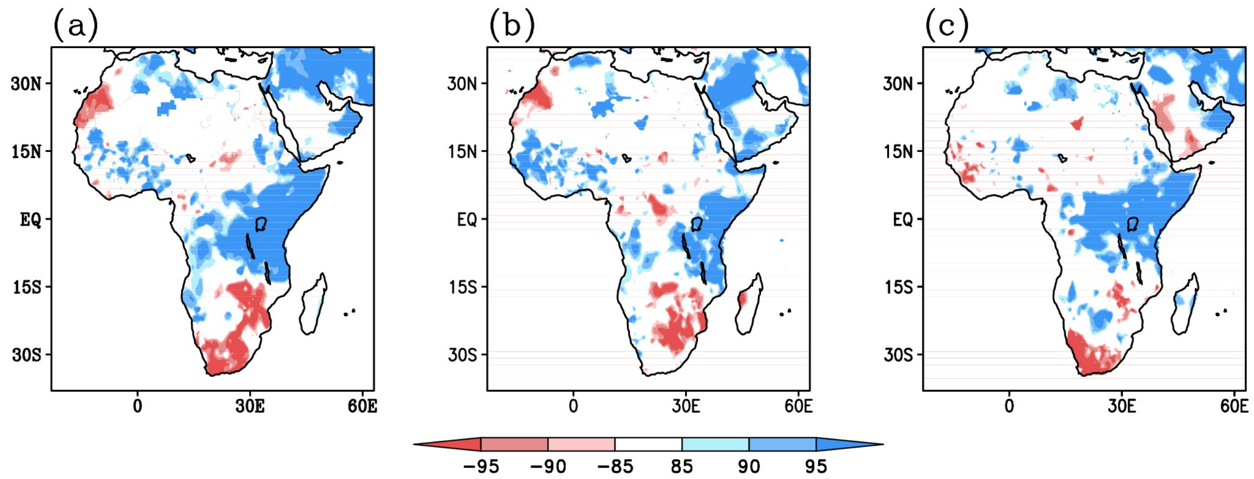

Figure A4. (a) Linear correlation between OND rainfall and IODMI. (b) Partial correlation between OND rainfall and IODMI, on removal of the influence from IOD western pole. (c) same as (b) but on removal of influence from IOD eastern pole. Correlations significant at 85%, 90% and 95% confidence level, based on Student's t-test are shown. [Figure created using the COLA/GrADS software].

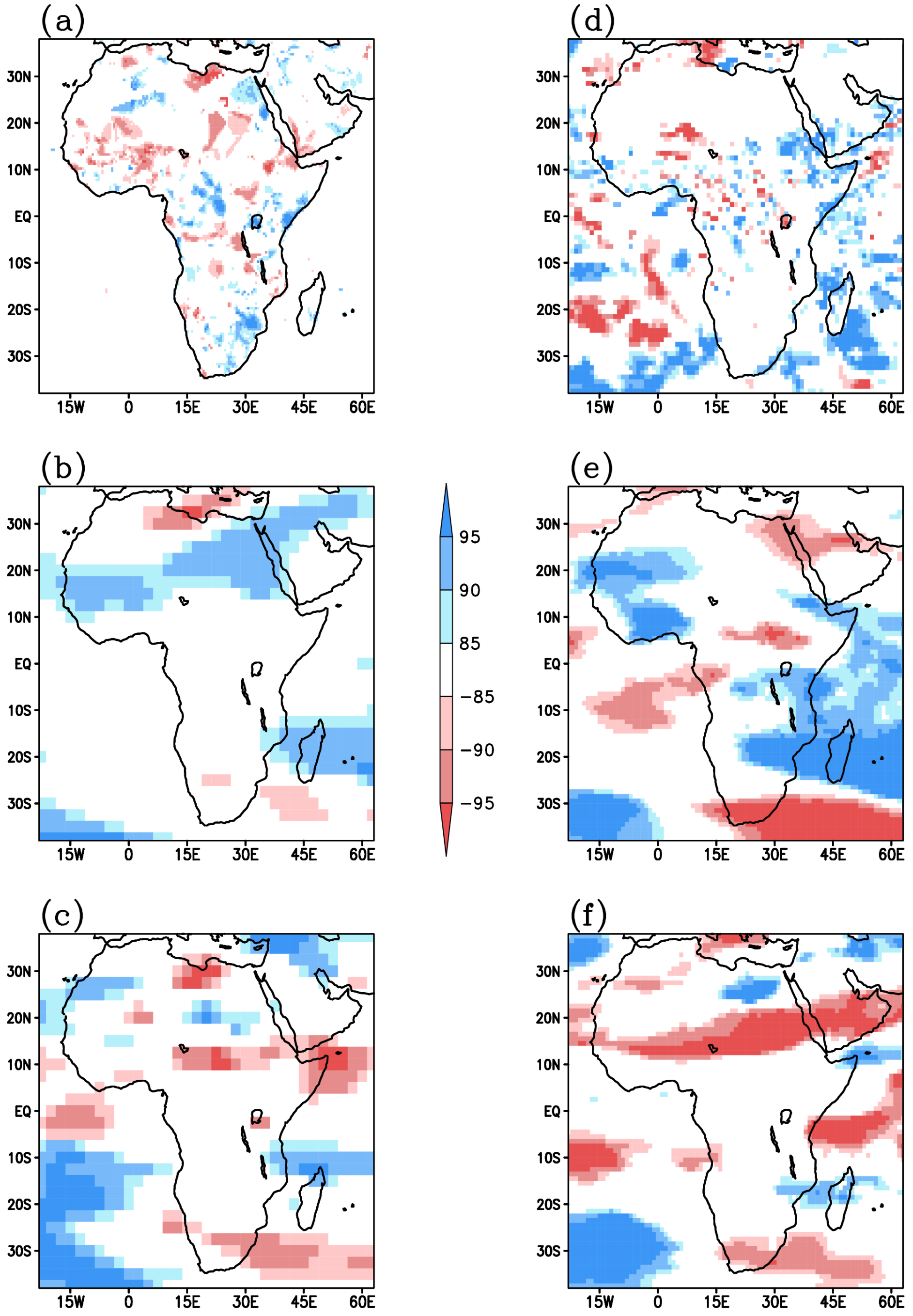

Figure A5. Difference between the mean JJAS anomalies, obtained from composite analysis, of (a) rainfall, (b) 200 hPa zonal wind and (c) 700 hPa zonal wind, associated with the canonical El Niño and that during El Niño Modokis. The corresponding difference between the canonical El Niño experiment and the El Niño Modoki experiments are shown in panels d, e and f. Significant values at 85%, 90% and 95% confidence level based on Student's t-test are shown in shadings. [Figure created using the COLA/GrADS software].

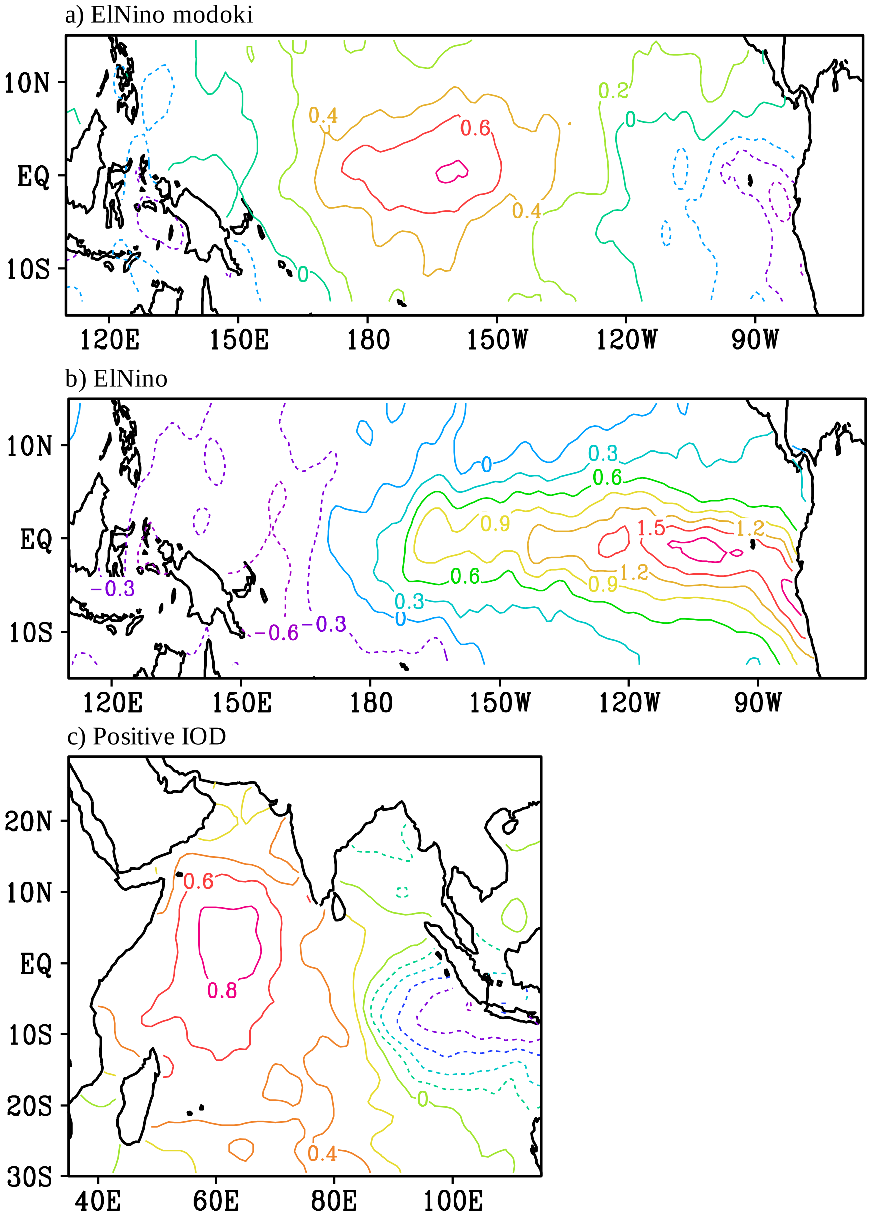

Figure A6. The imposed SSTA during September used as boundary forcing to the (a) El Niño Modoki, (b) El Niño and (c) positive IOD experiments. [Figure created using the COLA/GrADS software].
